# EHR-derived cognitive load is associated with guideline-concordant statin initiation in primary care

**DOI:** 10.1186/s12911-026-03392-6

**Published:** 2026-04-02

**Authors:** Ratnalekha V. N. Viswanadham, Yuhan Betty Cui, Priyanka Solanki, Nicole Redfern, Amelia Shunk, Angela Mastrianni, Defne L. Levine, Devin M. Mann, Safiya I. Richardson

**Affiliations:** 1 Department of Population Health, NYU Grossman School of Medicine, 227 E. 30th St, Sixth Floor, New York, NY 10016 USA; 2 Department of Medicine, NYU Langone Health, New York, NY 10016 USA; 3 Medical Center Information Technology, Department of Health Informatics, NYU Langone Health, New York, NY 10016 USA; 4 Tulane University School of Medicine, New Orleans, LA USA

**Keywords:** Statin, Clinical decision support, EHR, Process mining, Cognitive load, Audit log data, Machine learning

## Abstract

**Introduction:**

Linking electronic health record (EHR) use to care quality may offer insights into potential interventions improving guideline adherence and closing care gaps. We examine how EHR metadata can measure cognitive load in primary care providers during statin prescribing and identify cognitive load points in EHR workflows associated with guideline-concordant statin initiation.

**Methods:**

We retrospectively extracted 2024 data from EHR primary care encounters from a large academic health system. We identified adult patients who met the criteria for statin initiation and calculated their atherosclerotic cardiovascular disease (ASCVD) risk scores. Cognitive load metrics were derived from EHR metadata. Logistic regressions evaluate associations between cognitive load and statin initiation, adjusting for patient covariates and provider fixed effects. Gradient-boosted forests and Shapley Additive explanations (SHAP) values were used to identify key EHR events and cognitive load patterns associated with statin initiation.

**Results:**

Longer encounter duration was associated with increased likelihood of statin initiation, whereas more time spent per EHR event was associated with a decreased likelihood. Nonlinear associations were observed for loop count and distinct event count: predicted initiation probability decreased with increasing loop count to 93.9 loops, then increased beyond this threshold. For distinct events, initiation probability increased up to approximately 18 events and declined at higher counts. In a gradient-boosted decision tree model, average event time was the strongest predictor (72.2% relative contribution). Additional positive predictors included time spent reviewing lab results and on suggested medication order sets. Order list modification and looping back to it were negatively associated with statin initiation.

**Discussion:**

EHR metadata can associate cognitive load with appropriate clinical behavior, revealing nonlinear associations between cognitive load and statin initiation rates. This work suggests opportunities to optimize EHR systems to reduce cognitive burden and support clinical decision-making. Connecting cognitive load to prescribing behavior generates hypotheses about how workflow adjustments and enhanced decision support might improve guideline adherence and patient care through prospective evaluation.

**Supplementary Information:**

The online version contains supplementary material available at 10.1186/s12911-026-03392-6.

## Introduction

### Research goals and hypotheses

The objectives of this study were to assess how electronic health record (EHR) metadata can be used to measure cognitive load in primary care workflows for initiating guideline-concordant statin therapy. We pursued two aims. First, we evaluated whether cognitive load metrics were associated with the likelihood of appropriate statin initiation. Second, we explored whether specific EHR workflow events could be linked to cognitive load patterns that predict statin initiation. We hypothesized that providers who adhered to statin guidelines would exhibit greater cognitive effort and distinct EHR interaction patterns than those who did not. We expected this increased cognitive load to manifest as: (a) repeated actions to gather necessary information, (b) longer time spent per EHR activity, (c) more overall actions within the EHR, and (d) extended encounter duration. The second aim was exploratory, to identify which specific EHR events and their associated cognitive-load metrics might represent promising candidate targets for workflow redesign and future clinical decision support interventions, to be evaluated prospectively.

### Literature contribution (Table 1)

**Table 1:** Literature contribution table

Problem or issue	How can electronic health record (EHR) metadata identify ways to close clinical care gaps? What are EHR workflow cognitive load differences between providers who give appropriate care and providers who do not?
What is already known	Statins reduce cardiovascular risk but are under prescribed despite guidelines. Clinical decision support systems aim to improve prescribing but have low adoption. Cognitive load in providers can lead to errors and decision delays. EHR metadata captures clinician behavior and cognitive load, but few studies link it to specific clinical actions.
What this paper adds	A use case to associate EHR metadata metrics with care quality in healthcare.
Who would benefit from the new knowledge in this paper	Healthcare systems aiming to leverage EHR metadata to improve healthcare delivery. Health informaticians interested in leveraging data-driven approaches to improve clinical workflows. Behavioral scientists interested in understanding process workflows and sources of cognitive load in workflows. Policymakers improving quality of care through targeted interventions in EHR design and clinical guidelines.

## Related work

### Clinical context: statin prescribing for ASCVD risk prevention

Atherosclerotic cardiovascular disease (ASCVD) remains the leading cause of mortality globally, and failure to appropriately prescribe statins represents a missed opportunity for primary prevention at a population level [1]. Statins reduce the risk of major adverse cardiovascular events and mortality [2]. However, primary care providers fail to prescribe statin therapy for about half of patients meeting guideline criteria for initiation [3–8]. Among patients prescribed a statin, about two-thirds receive a lower-than-optimal dose [5]. To ensure patients are appropriately prescribed statins, providers must recognize that a patient meets one of four indications, often requiring calculation of a patient’s ASCVD risk and prescribing a guideline-approved statin at the appropriate dose. Individualized risk assessment requires collecting elements from the patient’s medical history, vital signs, and laboratory results, and then using a risk calculator. The EHR offers opportunities to develop clinical decision support systems (CDSSs) that support the recognition, assessment, and management of ASCVD risk, such as alerts and pre-populated medication orders. However, generally low provider adoption has limited the clinical impact of current CDSSs designed to improve guideline-concordant statin prescribing [9–13]. Additionally, evolving guidelines and varying thresholds for treatment based on age and comorbidities further complicate the prescribing process, especially without automated support.

### EHR metadata to evaluate EHR utilization

Cognitive load can influence the quality of healthcare decision-making by increasing the likelihood of delayed action, reliance on cognitive shortcuts, and misalignment with evidence-based care [14, 15]. In the EHR environment, poorly integrated workflows and excessive demands on attention have been associated with clinician burnout and suboptimal patient outcomes [16]. Quantifying cognitive load in routine practice is therefore critical for understanding its role in decision-making and identifying opportunities for improvement [17]. Traditional approaches to studying clinician-EHR interactions, such as time-and-motion studies, direct observation, surveys, and interviews, are resource-intensive and prone to biases, such as the Hawthorne Effect, in which awareness of being observed alters behavior [18]. These methods may also fail to capture subtle but important real-time decision points.

EHR metadata is an automated tracking feature that logs system behavior (such as adding, deleting, or querying information) along with contextual information (timestamp, user, and patient). EHR certification mandates its tracking for security and privacy purposes. As of 2017, 96% of hospitals and 80% of private practices that used EHRs were compliant with these criteria [19]. Specifically, EHR audit log metadata, collected for HIPAA compliance to track what providers view and click in the EHR, can serve as a non-invasive method to observe how providers and patients interact within the EHR without imposing additional burdens on clinicians [20]. For instance, audit log data can be used to measure how many times a provider clicks between the lab results and medication order screen during a visit, offering a proxy for task-switching or uncertainty. Using EHR metadata provides an analytical approach that not only captures natural interaction patterns but also enables scalable, objective measurement of cognitive load and its associations with prescribing behavior, laying a foundation for future interventions that can be assessed in real-world practice patterns.

EHR audit log metadata offers expanding opportunities for health services research [21]. The clinical informatics literature has categorized metadata metrics that directly capture EHR user behavior related to cognitive load [22]. However, extracting and interpreting these metrics to meaningfully reflect cognitive load is technically challenging. Raw EHR audit log metadata are high-volume, inconsistently structured across systems, and not collected with research in mind, making it challenging to align events with specific clinical decision-making moments. While the literature is limited in linking these metrics to specific healthcare research questions and in measuring the effectiveness of healthcare delivery [23], prior work has shown that process mining and related event-log methods can be useful to transform raw audit-log events into clinically meaningful measures of workflow and behavior. In this study, we adopt this general paradigm by deriving encounter-level cognitive-load metrics from raw audit-log events and relating them to statin initiation; the extraction and processing steps are described in detail in Methods (“Data and Data Structure”).

This study aims to fill a critical gap by describing how EHR-derived cognitive-load metrics differ between encounters in which eligible patients do and do not receive a statin, providing insight into workflow patterns that may be relevant to closing care gaps in ASCVD prevention. Additionally, we aim to identify which EHR activities (e.g., medication orders, patient communications, and the use of pre-existing tools) are most strongly associated with a higher cognitive load in encounters that result in guideline-concordant statin prescribing. These objectives are exploratory and focus on characterizing associations rather than establishing causal effects of cognitive load on prescribing behavior.

## Methods

We conducted a retrospective, cross-sectional, observational study using EHR audit log metadata and clinical encounter data to evaluate the association between cognitive load during primary care visits and initiation of statin therapy. This study was designed and reported in accordance with the Strengthening the Reporting of Observational Studies in Epidemiology (STROBE) guidelines [24]. This study was approved by the NYULH Institutional Review Board (IRB #i23-01349), which granted a waiver of authorization.

### Population and number of observations

Data were retrospectively pulled from the EHR of a large academic health system that uses Epic Systems between January 1, 2024, and December 31, 2024, for primary care encounters of patients who qualified for statin initiation during their clinic visit.

### Data inclusion and exclusion

To determine statin eligibility for adult patients between 20 and 75 years of age, we extracted laboratory data, including their most recent lipid panels and hemoglobin A1c, age, self-identified race and ethnicity, sex, smoking history, latest collected systolic blood pressure, medication history, and any previous history of ASCVD events to calculated each patient’s ASCVD risk based on the 2013 American College of Cardiology/American Heart Association (ACC/AHA) ASCVD 10-year risk score [25]. We identified each eligible patient’s most recent primary care visit in 2024 and retrieved encounter-level metadata, including provider identifiers.

We first identified adult patients with at least one documented primary care encounter in 2024. Patients were excluded if they had no primary care encounters during the study period, indicated an allergy or contraindication to a statin, or were pregnant in 2024. Patients were also excluded if they were missing one or more variables required to compute the 2013 ACC/AHA 10-year ASCVD risk score (age, sex, systolic blood pressure, total cholesterol, HDL, LDL, diabetes status, and smoking status) within 3 years preceding their primary care encounter, consistent with how these data are routinely captured and 10-year ASCVD risk calculated in the health system’s EHR [25]. Patients who qualified for statin therapy were identified based on the guidelines in Table 2 for primary and secondary prevention, which are based on the ACC/AHA Task Force on Clinical Practice’s guidelines on the management of blood cholesterol [26]. For this study, we pragmatically pooled all guideline-based indications into a single “statin-eligible” category rather than analyzing each indication as a separate stratum. This approach reflects how clinicians encounter overlapping indications in routine practice, ensures adequate sample size for stable estimation, and allows us to focus on overall cognitive load differences between eligible encounters. Patients were then classified based on their statin prescribing status at their latest visit: initiated, defined as those with a statin medication order documented during the encounter, and not prescribed, defined as those without a corresponding statin order despite meeting guideline-based indications. Statins initiated in 2024 are listed in Table A1.

**Table 2:** Inclusion and exclusion criteria for Statin therapy eligibility

Inclusion Criteria	Exclusion Criteria
Patients with any of the following guidelines: • Age 20 to 75, with LDL ≥ 190 mg/dL and diagnosis of Type 2 Diabetes Mellitus^1^ • Age 40 to 75, with Type 2 Diabetes Mellitus^1^ and a history of myocardial infarction (MI)^2^ or stroke^2^ • Age 40 to 75, with an estimated 10-year ASCVD risk ≥ 7.5%^3^ • Age 20 to 75, with a history of myocardial infarction (MI)^2^ or stroke^2^	Patients with any of the following conditions: • Pregnant • Have a known allergy to statins or a contraindication to statin use • Have any of the following diagnoses: o Hepatitis^2^ o Cirrhosis^2^ o End-Stage Renal Disease (ESRD)^2^ o Chronic Kidney Disease (CKD)^2^

^1^Type 2 Diabetes Mellitus identified via ICD-10 codes (Table A.2.) or HbA1c ≥ 6.5% in their latest blood draw

^2^ Diagnoses identified using ICD-10 codes listed in Table A.2

^3^ 10-year ASCVD event risk was measured using the 2013 ACC/AHA guidelines [25]

### Data and data structure

EHR audit log metadata and relevant patient clinical information were extracted from the healthcare system’s data lake, built on a Hadoop distributed file system, and queried using MySQL databases. For patients initiated on statins in 2024, we extracted metadata from the encounter linked to the initiation. For those not initiated, we used metadata from their most recent annual wellness visit, which occurred in 2024. A primary care encounter was identified by a single contact serial number (CSN) in the EHR data lake and defined as a clinical contact with a patient with scheduled patient interaction. Because work on an encounter can occur in several sessions, a given CSN may consist of multiple, non-contiguous time segments during which the provider actively engages with the patient’s data or performs related actions. For example, a provider might review the patient’s chart regarding an encounter from 1:00 PM to 1:03 PM, conduct a face-to-face visit from 1:40 PM to 2:10 PM, and later complete documentation after work hours (between 7:00 PM and 7:00 AM) or even the next day. The provider’s encounter duration would be the sum of their active, non-contiguous time segments associated with the CSN.

For each provider-encounter (provider ID-CSN) pair, we ordered all audit log events chronologically and grouped them into active time segments. The encounter start time for that provider-CSN pair was defined as the timestamp of their first event with that CSN. The encounter end time was defined as the timestamp of their last event with that CSN plus the minimum of (a) the elapsed time until the next event for a different CSN or (b) a 10-minute inactivity timeout, to account for idle time when the record may have been left open without active work. All active segments for a provider-CSN pair were then combined to obtain the provider-specific total active encounter time. If the same event type occurred sequentially (for example, multiple consecutive “Chart viewed” actions), we aggregated them into a single event, summing their active time, to reduce spurious inflation of event duration caused by very fine-grained logging of the same screen remaining open. Multiple statin-prescribing providers (physicians, nurse practitioners [NPs], and physician assistants [PAs]) could contribute events under the same CSN; thus, the unit of analysis for the regression analysis is the provider-encounter pair.

From the provider-encounter-level audit logs, we derived four cognitive load metrics based on the user interactions (“events”) logged by the EHR: (1) the number of loops (revisits to a previously opened event), (2) the number of distinct EHR events accessed, (3) the total active encounter duration in minutes, and (4) the average time spent per EHR event in seconds (Table 3). In this work, “cognitive load” is approximated using four audit log metrics, which capture aspects of effort and workflow complexity but do not encompass all dimensions of cognitive workload. EHR events include instances when a provider opens, reviews, or edits patient data (e.g., laboratory results, problem lists, medication orders), as well as other workflow activities associated with the same CSN and provider ID. The ordered sequence of timestamped events allows us to summarize how intensively and broadly each provider interacted with the record during the statin-eligible visit. Each metric was chosen because it represents features that can, in principle, be modified through workflow or interface changes (for example, reducing unnecessary loops or consolidating related tasks), making these metrics candidate targets for future decision-support and EHR design change interventions evaluated prospectively.

**Table 3:** Cognitive load metric definitions

Cognitive Load Metric	Definition	Example of Cognitive Load Metric	Associations between Metric and Opportunity for Behavior Change
Number of Loops (N)	The number of times an event is revisited during an encounter.	User moves from Event A → Event B → Event C → Event A → Event B → Event D (2 loops total – one loop back to Event A and one loop back to Event B)	More loops could increase cognitive effort due to redundancy or confusion, suggesting redesigning workflows to remove unnecessary repetition.
Average Time Spent on an EHR Event (seconds)	The average time spent on an EHR event.	8.7 s per event (e.g., Event A, Event B, Event C)	More time spent per event indicates complexity and more fixation, suggesting opportunities for simplification or support.
Number of Distinct EHR Events	The number of unique events in the EHR that a provider visits during their encounter.	15 unique events (Event A, Event B, Event C, …, Event O)	More distinct events increase task complexity and intrinsic load. More distinct events suggest chunking related tasks or adding grouping mechanisms.
Duration of an Encounter (minutes)	The total active time an agent spends engaging with data or actions related to a specific customer interaction, summed across all non-contiguous time segments associated with the same encounter ID.	17.9 min from first event (A) to last event (C) related to an encounter	Longer encounter duration may indicate more time engaging with the EHR for reviewing patient data, which could introduce fatigue or signal focused patient engagement. Shorter encounter duration may be related to more goal-oriented behaviors and lower cognitive load.

### Analytical methods

All analyses were done in RStudio Version 2024.12.0 + 467.

#### Descriptive statistics

We collected means, standard deviations, medians, and ranges of cognitive load metrics, patient characteristics during their encounter with the provider, and provider sex. We used the interquartile range (IQR) method to identify potential outliers in measures of EHR interaction complexity, including the number of loops, the number of distinct events, the total duration, and the average time per event. Following the Tukey IQR rule [27], values falling more than 1.5 times the IQR below the first quartile or above the third quartile were classified as outliers. A record was flagged as an outlier if any cognitive load metric exceeded these thresholds.

#### Associations between statin prescribing and cognitive load metrics

The primary outcome of interest was whether a statin was initiated during a statin-eligible encounter by a provider (yes/no). We modeled this outcome using logistic regression analysis, with each observation representing a stain-prescribing provider-encounter pair. The main independent variables were the four encounter-level cognitive-load metrics derived from the audit log: number of loops, average time spent per EHR event, number of distinct EHR events, and encounter duration (Table 3). To adjust for case complexity and patient mix, we included patient-level covariates for sex, self-identified race and ethnicity, insurance status (commercial versus non-commercial), Elixhauser Comorbidity Index [28] for patient case complexity during their visit, the number of active diagnoses during their encounter, and age. We incorporated provider fixed effects to control for unobserved, time-invariant characteristics of each provider (for example, baseline prescribing tendency or years of experience), so that estimates are identified from within-provider differences across encounters. Because multiple statin-prescribing clinicians (physicians, NPs, and PAs) can contribute events under the same encounter identifier, we used cluster-robust standard errors at the encounter level to account for residual correlation within encounters [29]. 

Cognitive load metrics may not relate linearly to the probability of initiating a statin; low or moderate effort can sometimes support high-quality, streamlined decision-making, whereas very high effort may reflect complexity, fragmentation, or difficulty completing the task [30–32]. In other cases, clinical decision-making improves as cognitive challenge or task engagement increases, up to a saturation point, after which it declines, suggesting an optimal “middle ground” of effort. In other cases, low-effort task sequences may reflect automation of desired behaviors, whereas high-effort sequences may signal increased deliberation. In contrast, certain intermediate levels of effort could lead to inertia, resulting in no action. To accommodate non-linear patterns, we added quadratic terms for each cognitive load metric to flexibly capture potential threshold effects. We tested whether excluding the quadratic terms would significantly affect model fit with a Wald test between the linear and nonlinear models [33]. If excluding the nonlinear term did not significantly affect model fit, the quadratic term was removed to reduce the risk of overfitting. For metrics with retained quadratic terms, we computed average marginal effects across the observed range to summarize how the predicted probability of statin initiation changed as the metric increased. This approach enabled us to quantify both the direction and magnitude of the relationship across different levels of cognitive load, identifying points at which additional effort began to hinder rather than support statin initiation. We report the area under the receiver operating characteristic (ROC) curve (AUROC) as a measure of model fit [29]. To test whether extreme values drove estimates, we performed sensitivity analyses excluding the top and bottom 1% of each cognitive load metric to assess changes in the coefficients [34]. 

#### Exploratory analyses – sources of cognitive load within the EHR

We aimed to link cognitive load measures within the EHR to statin initiation to identify opportunities for integrating clinical decision support. Using machine learning, we identified which EHR events were most strongly associated with statin initiation and the types of cognitive load associated with those events. We structured the EHR metadata of a patient’s entire encounter so that each variable represented a cognitive load metric for a specific EHR event. This means, for example, we measured the average time a provider spent at Event A, how many times a provider looped back to Event B, and whether the provider visited Event C within an encounter. With around 215 possible EHR event types and 3 cognitive load metrics (time spent, loops, and visiting the event), the final feature set included approximately 645 event-cognitive load variables. Because not every provider interacts with every EHR event, many feature values were zero, reflecting events that were skipped or lacked in the cognitive load metric. Each row of the dataset corresponded to a single encounter ID rather than a provider–encounter pair because fixed effects are not readily implemented in the machine learning framework. To partially account for provider mix, we included indicator variables for whether a physician, NP, or PA participated in the encounter. We also included patient-level covariates from the logistic regressions to account for the complexity of the patient case and other patient factors that could be significantly associated with statin initiation.

To explore how these detailed interaction patterns relate to statin initiation, we used an extreme gradient-boosted decision tree model that took these variables as inputs to predict whether a statin was initiated during the encounter using the XGBoost package in R [35]. We selected this method because our modeling dataset is high-dimensional and sparse, with hundreds of event-level cognitive load features and many provider-encounter observations; gradient-boosted forests are well-suited for such “wide” tabular data, handle nonlinear relationships and interactions without pre-specifying functional forms, and naturally accommodate missing or zero-inflated event metrics. This approach allowed us to identify which specific EHR events’ cognitive-load metrics were most strongly associated with the decision to prescribe a statin, rather than imposing strong linearity or additivity assumptions.

We used a repeated train–validation–test framework to estimate and evaluate the XGBoost models. Specifically, for each run, we randomly partitioned the analytic dataset at the encounter level into 70% training, 15% validation, and 15% test sets. The training set was used to fit candidate models, the validation set was used for hyperparameter tuning and early stopping, and the held-out test set was reserved exclusively for final performance assessment. Within each run, we trained XGBoost models with a logistic objective. We optimized AUROC on the validation set using up to 300 boosting iterations, with early stopping after 10 rounds without improvement in validation AUROC. The model with the best validation AUROC was then applied to the test set to obtain predicted probabilities and compute the test AUROC. To obtain stable estimates and quantify variability, we repeated this 70-15-15 split and training procedure 200 times with different random seeds.

To interpret the model outputs, we applied Shapley Additive explanations (SHAP) values, which attribute feature contributions to individual predictions in tree-based models. SHAP values provide local interpretability (how specific features influence a given prediction) and global interpretability (the overall importance of features across the dataset) [36]. We calculated the mean SHAP values for each feature and the global mean SHAP value (the mean of the absolute value of the SHAP values) to summarize both the direction (negatively or positively contributing to the prediction, as per the sign of the mean SHAP value) and the magnitude of each event-level cognitive-load metric. These summaries allowed us to identify EHR activities whose cognitive-load metrics were most strongly associated with statin initiation, generating hypothesis-generating information about potential workflow leverage points for future decision-support interventions. Significant features were defined as those for which the 95% confidence interval of the mean SHAP value did not contain 0. Features that were not significant or that had SHAP values of 0 were filtered from analyses. For each feature, we defined its relative importance as its global mean SHAP value divided by the sum of the global mean SHAP values across all significant features, and features were then ranked from the largest to the smallest relative contribution. Summarizing relative importance in this way allows comparing the contributions of cognitive‑load metrics, patient covariates, and other EHR activity features on a common scale, clarifying how strongly workflow‑related cognitive load was associated with model‑predicted statin initiation relative to traditional clinical factors [37]. 

In sensitivity analyses to assess potential target leakage (e.g., the significance of post-prescribing events as artefacts in the SHAP importance due to the use of whole-encounter audit logs), we re-estimated models and SHAP values after truncating initiated encounters at the statin order time and non-initiated encounters using a windowing scheme that truncated encounters at either the first order screen or the median prescribing time from the initiation encounters if medication ordering was absent from the non-initiation encounter. The differences between the whole model and the sensitivity analyses can be found in Table 4.

**Table 4:** Truncation rules and analytic samples for the primary and pre-decision XGBoost models used in SHAP analysis, showing how encounters were censored to include only pre-decision events and which post-decision events were excluded

Model	Encounter Type	Truncation Rule (End Time)	Events Excluded	Encounter (*N*)
Full-encounter XGBoost	Initiation Encounters and Non-Initiation Encounters	No truncation (all audit log events in encounter)	None	20,373
Pre-decision XGBoost sensitivity	Initiation Encounters	First statin order timestamp	All events after statin order	9271
Pre-decision XGBoost sensitivity	Non-initiation encounters	First medication order screen; If no medication ordered, median time to statin order from initiated visits	All events after the truncation time.	11,052 (first medication order screen)
				50 (no medication ordered)

## Results

### Descriptive results

We analyzed 20,376 patient encounters across the academic healthcare system. The mean age of patients eligible for statin initiation was 58.8 years (SD = 10.8), and about half were male. Three patients were of unknown sex and excluded from the reporting and analysis [38]. The cohort was predominantly non-Hispanic/Latinx White, and approximately 80% had commercial insurance. Each patient had an average of 6 active diagnoses during their primary care provider encounter. Most encounters were handled by physicians (82.41%), with fewer by nurse practitioners (NPs, 11.66%) and physician assistants (PAs, 5.93%). Encounters with NPs had the highest statin initiation rate (50.72% of NP encounters resulted in a statin initiation) compared to 46.86% for physicians and 46.6% for PAs. Encounters with statin initiation showed slightly lower cognitive load metrics on average than encounters without statin initiation. These differences were small but statistically significant: encounters with initiation had fewer loops, fewer distinct events, shorter duration, and a lower average time per event.

**Table 5:** Descriptive statistics of patients, providers, and cognitive load metrics

	No Initiation	Initiation	*P*-value
	(*N* = 11102 Patients)	(*N* = 9271 Patients)	
**Patient Characteristics**			
**Age (years)**			
Mean (SD)	58.9 (10.8)	58.7 (10.8)	0.213
Median [Min, Max]	61.0 [20.0, 75.0]	60.0 [20.0, 75.0]	
**Sex**			
Male	5542 (49.9%)	4788 (51.6%)	0.0141
Female	5560 (50.1%)	4483 (48.4%)	
**Race Ethnicity**			
Non-Hispanic/Latinx White	6609 (59.5%)	4366 (47.1%)	< 0.001
Non-Hispanic/Latinx Black/African American	1434 (12.9%)	1098 (11.8%)	
Hispanic/Latinx	1091 (9.8%)	1791 (19.3%)	
Asian	844 (7.6%)	723 (7.8%)	
Other	1124 (10.1%)	1293 (13.9%)	
**Financial Class**			
Commercial	8904 (80.2%)	7263 (78.3%)	0.00111
Non-commercial	2198 (19.8%)	2008 (21.7%)	
**Number of Active Diagnosis**			
Mean (SD)	6.40 (3.07)	6.09 (3.51)	< 0.001
Median [Min, Max]	6.00 [1.00, 24.0]	5.00 [1.00, 32.0]	
**Elixhauser Comorbidity Index**			
Mean (SD)	1.68 (5.42)	1.42 (5.37)	< 0.001
Median [Min, Max]	0 [-14.0, 42.0]	0 [-18.0, 58.0]	
**Number of Providers Involved in Encounter**			
One	8132 (73.2%)	6866 (74.1%)	
Two	2635 (23.7%)	2154 (23.2%)	
Three	335 (3.0%)	251 (2.7%)	
**Provider Type**	**Physician**	**Nurse Practitioner**	**Physician Assistant**
Number of Encounters	33,277 (82.42%)	4706 (11.66%)	2391 (5.92%)
**Initiation Rate**			
Mean (SD)	46.86 (0.77)	50.88 (1.47)	46.61 (2.00)
Median [Min, Max]	46.15 [0, 100]	50.00 [0, 100]	41.05 [0, 100]
	**No Initiation**	**Initiation**	
**Cognitive Load Metrics**	**(N = 11102 Encounters)**	**(N = 9271 Encounters)**	**P-value**
**Number of Loops**			
Mean (SD)	75.0 (34.2)	71.1 (39.4)	< 0.001
Median [Min, Max]	70.0 [1.00, 311]	63.0 [0, 465]	
**Number of Distinct Events**			
Mean (SD)	34.0 (6.01)	33.4 (7.00)	< 0.001
Median [Min, Max]	34.0 [8.00, 69.0]	33.0 [9.00, 80.0]	
**Encounter Duration (Minutes)**			
Mean (SD)	38.8 (20.0)	37.8 (22.1)	< 0.001
Median [Min, Max]	35.0 [1.17, 183]	33.0 [0.333, 246]	
**Average Time per EHR Event (Seconds)**			
Mean (SD)	44.5 (61.3)	40.9 (55.9)	< 0.001
Median [Min, Max]	22.7 [1.84, 970]	22.0 [1.11, 791]	

Overall, 17.6% of records had at least one extreme value in the cognitive load metrics (flagged as outliers per the Tukey IQR rule). A chi-squared test showed no significant difference in statin initiation rates between encounters with vs. without such outlier metrics (*p* < 0.20): 45.7% versus 44.5% initiation, respectively. Risk analysis revealed that outlier encounters were 3% less likely to involve statin initiation (RR = 0.97; OR = 0.95, 95% CI 0.89–1.03), with no significant difference in statin prescribing likelihood between the two groups. We therefore retained all observations in subsequent analyses. Descriptive analyses are presented in Table 5.

### Regression analyses between cognitive load metrics and statin initiation

We examined whether the cognitive load metrics were associated with the likelihood of statin initiation in a multivariate logistic regression model, adjusting for patient characteristics and provider effects. Because the unit of analysis is the provider-encounter pair, encounters with multiple statin-prescribing providers contribute multiple observations; we used provider fixed effects and encounter-level cluster-robust standard errors to address within-provider and within-encounter dependence. Initially, we tested a model with only linear terms for each cognitive load metric; however, two metrics—encounter duration (in minutes) and the average time spent per EHR event (in seconds)—did not show a significant linear relationship with statin initiation. We then examined non-linear relationships by adding quadratic terms. Model comparison revealed that quadratic terms for the number of loops and the number of distinct events significantly improved the fit (Wald test, *p* < 0.001). Thus, our final model included non-linear (quadratic) terms for loops and distinct events, as well as linear terms for duration and time per event. Comparisons of the linear and nonlinear models are presented in Table A.3.

#### Linear effect of cognitive metrics on statin initiation

Regression results are presented in Table 6. In the final regression model, the average time spent per EHR event and the total encounter duration were significantly associated with statin initiation. The average time spent per event showed a significant negative association with the likelihood of statin initiation, where spending more time per event was associated with a lower likelihood of initiation (β = -5.017 × 10^− 4^, *p* < 0.001). In contrast, the total encounter duration showed a significant positive association with the odds of initiating a statin, with longer duration associated with higher odds (β = 8.223 × 10^− 3^, *p* < 0.001). These associations may reflect provider time-management strategies, in which extended engagement across multiple sections of the EHR is more often observed in encounters where guideline-recommended treatment is initiated, although causal mechanisms cannot be inferred from these observational data.

#### Nonlinear effects of cognitive metrics on statin initiation

**Fig. 1:**
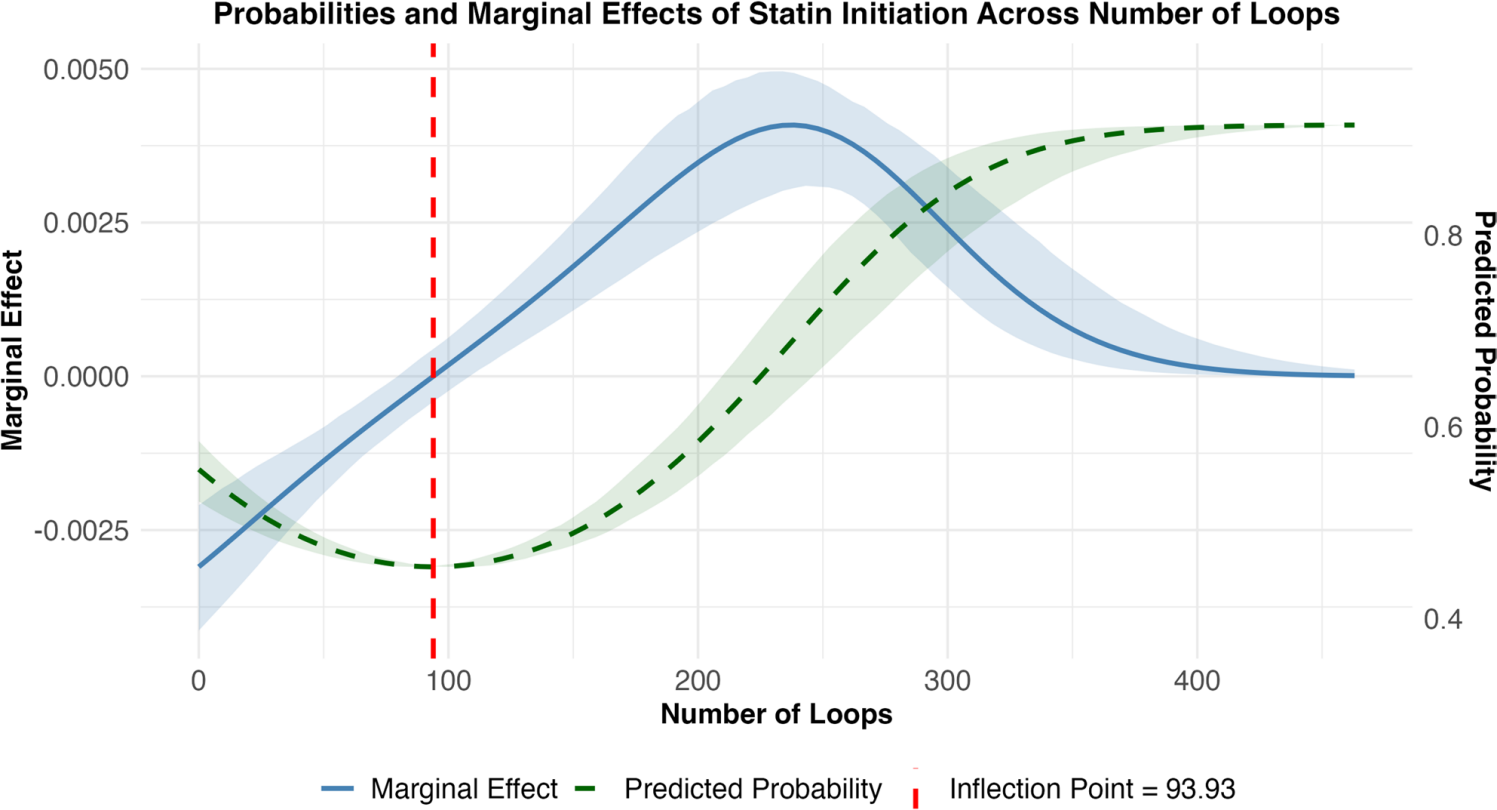
Plotted predicted probabilities and marginal effects of initiating a statin relative to the number of EHR event loops

**Fig. 2:**
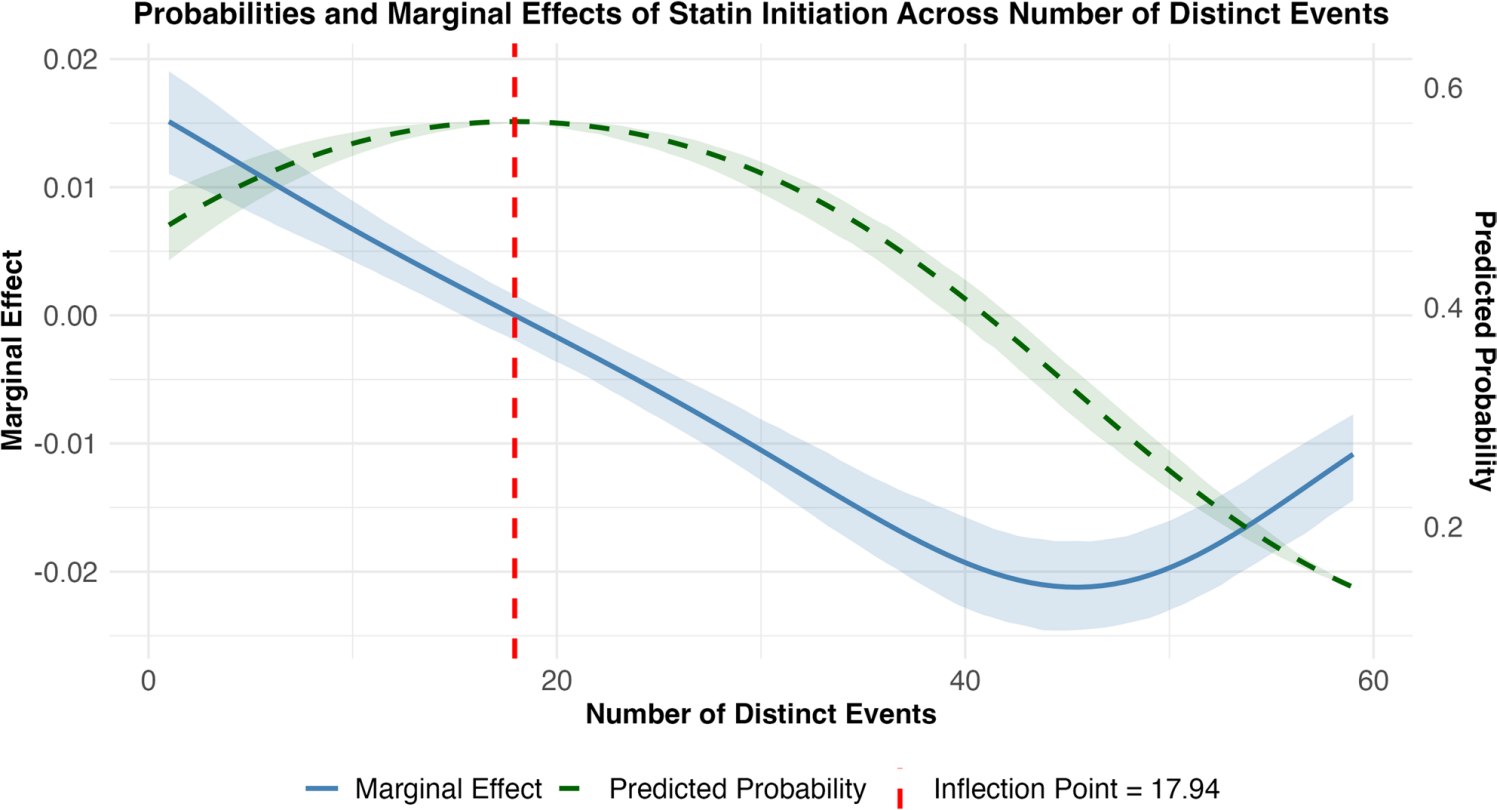
Plotted predicted probabilities and marginal effects of initiating a statin relative to the number of distinct EHR events

Figures 1 and 2 illustrate the two cognitive load metrics that exhibited significant nonlinear associations with statin initiation. For the number of loops, coefficients indicated a U-shaped relationship (linear β = −0.0124, *p* < 0.001; quadratic β = 0.597 × 10⁻⁵, *p* < 0.001). Encounters with either very low or very high loop counts were more likely to be associated with statin initiation than those with intermediate counts (Fig. 1, dashed green line). The inflection point occurred at 93.9 loops (Fig. 1, vertical dashed red line); below this threshold, each additional loop was associated with a 0.21% lower predicted probability, whereas above it, each additional loop was associated with a 0.00046% higher predicted probability. The peak effect was observed at 237 loops, with a predicted initiation probability of 68.4%.

The number of distinct events showed an inverse U-shaped relationship (linear model: β = 0.0124, *p* < 0.001; quadratic model: β = −0.597 × 10^− 5^, *p* < 0.001), with the highest probability of initiation observed when encounters have moderate event counts. The inflection point occurred at approximately 18 events (Fig. 2, vertical dashed red line); below this threshold, each additional event was associated with a 1.35% higher predicted probability of initiation, whereas above it, the effect was negligible, and the probability gradually declined. This pattern is consistent with an optimal range of distinct EHR actions that is associated with more frequent guideline-concordant statin initiation, with diminishing incremental gains beyond this threshold. These nonlinear shapes are descriptive associations and may also partly reflect unmeasured workflow and team-level factors, rather than causal effects of distinct events or prescribing.

Patient race and sex were also significant predictors of statin initiation, as shown in Table 6. Compared to non-Hispanic/Latinx White patients, patients of racial and ethnic minorities were more likely to be initiated on a statin, as well as older patients. Female patients, as well as those with a greater number of active diagnoses or higher comorbidity burden, were less likely to receive initiation.

We conducted four separate sensitivity analyses. In each model, we removed the top and bottom 1% of one cognitive load metric at a time and re-estimated the same logistic regressions. Across all four trimmed models, the direction and statistical significance of all workflow variables remained stable. The linear and quadratic terms for the number of loops and distinct events retained the same signs and similar magnitudes. Both encounter duration and average time per event also showed consistent associations with statin initiation. No variables changed sign, and all core workflow terms remained statistically significant. Demographic and clinical covariates exhibited similar stability. These findings indicate that extreme values do not drive the regression model results. These comparisons are presented in Table A.4.

**Table 6:** Coefficients of the nonlinear model associating cognitive load with statin initiation

	Reference Level	Coefficient	Std. Err	Statistic
Number of Loops	--	−1.239e − 02***	(2.123e − 03)	-5.84
Number of Loops²		6.597e − 05***	(1.021e − 05)	6.46
Number of Distinct Events	--	6.412e − 02***	(8.381e − 03)	7.65
Distinct Events²		−1.787e − 03***	(1.762e − 04)	-10.1
Total Duration (min)	--	8.223e − 03***	(1.436e − 03)	5.73
Avg Time Per Event (sec)	--	−5.017e − 04***	(1.314e − 04)	-3.82
Race: Black/African American	Non-Hispanic/Latinx White	1.967e − 01**	(6.567e − 02)	3
Race: Hispanic/Latino	--	5.906e − 01***	(6.508e − 02)	9.08
Race: Asian	--	4.425e − 01***	(8.083e − 02)	5.47
Race: Other		6.839e − 01***	(6.363e − 02)	10.7
Patient Age		1.023e − 02***	(2.101e − 03)	4.87
Patient Sex: Female	Male	−8.218e − 02*	(4.103e − 02)	-2
Insurance: Non-commercial	Commercial	5.738e − 02	(5.466e − 02)	1.05
Number of Active Diagnoses		−4.879e − 02***	(7.546e − 03)	-6.47
Elixhauser Comorbidity Index		−9.656e − 03**	(3.742e − 03)	-2.58
N Observations		36,131		
AUROC		0.784		

+ *p* < 0.1, * *p* < 0.05, ** *p* < 0.01, *** *p* < 0.001

### Exploratory analyses for EHR events associated with cognitive load metrics

We analyzed EHR metadata using a machine learning model to identify which specific EHR activities and associated cognitive load metrics were most strongly associated with statin initiation. We trained a gradient-boosted decision tree model and computed SHAP values to quantify the contribution of each EHR interaction feature to the model’s predictions. We conducted two analyses: one using cognitive load-event metrics estimated from full encounter audit log sequences, and, to assess artefacts from post-decision EHR interactions, a second using cognitive load-event metrics from encounters truncated at the statin order time in initiation or, in non-initiation encounters, the minimum of the first medication order or the median time of prescribing.

In the full-encounter XGBoost model, EHR workflow event features remained the dominant contributors to predictions of statin initiation, with an AUROC of 0.87 indicating a strong fit [39]. The top 10 contributing features accounted for about 60% of the summed absolute SHAP values, indicating that they contributed the majority of the model-attributed variation in predicted probabilities, relative to other features. Event-time metrics and two clinical covariates (number of active diagnoses and Hispanic/Latinx ethnicity) contributed to most of the importance (for top 10 features and Appendix Table A.5 for a full list of significant features). Average time per event accounted for nearly three-quarters of the total relative contribution across all significant features (72%) and much of the contribution among the top 10 features, followed by event presence (16%), covariates (8%), and the number of loops (4%).

Longer average time per event when the order list was changed, when a prebuilt order set (“SmartSet”) was suggested, when follow-up plans were modified, and when the prebuilt order set activity was open had a positive mean SHAP value, indicating that the higher values of these features were associated with higher model-predicted probabilities of statin initiation. The presence of a suggested SmartSet (event-show indicator) similarly had a positive mean SHAP value. In contrast, longer average time per event viewing In Basket messages and reviewing or acknowledging results in the inbox had negative mean SHAP values, indicating that more time on these inbox activities was associated with lower predicted probabilities of initiation, despite their large global contributions (i.e., higher global mean SHAP values). A higher number of active diagnoses and Hispanic/Latinx ethnicity also had positive mean SHAP values, indicating that these patient characteristics were associated with higher predicted probabilities of statin initiation in the model. These SHAP-based associations highlight patterns in how clinicians allocate time across inbox management, order-set use, and follow-up planning that are highly informative descriptive model associations (Table 7).

**Table 7:** Top 10 cognitive load features associated with Statin initiation in whole encounter audit log data, ranked by mean absolute SHAP value. The total relative contribution of these features is 60.39% of the model’s predictions

Rank	Metric Name	Plain Language Explanation	Cognitive Load Metric	Mean SHAP Value	Global Mean SHAP Value	95% CI Lower	95% CI Upper	Contribution Direction	Relative Contribution	Cumulative Contribution
1	In Basket message viewed	Inbox message viewed	Average Time Per Event (sec)	-0.0110	0.4638	-0.0041	-0.0179	Negative	12.82%	12.82%
2	Order list changed	Medication/order list changed	Average Time Per Event (sec)	0.0203	0.3935	0.0265	0.0141	Positive	10.87%	23.69%
3	SmartSet suggested	Prebuilt order set suggested	Average Time Per Event (sec)	0.0065	0.2742	0.0105	0.0025	Positive	7.58%	31.27%
4	Results acknowledged in IB	Test result acknowledged in inbox	Average Time Per Event (sec)	-0.0090	0.2238	-0.0057	-0.0124	Negative	6.18%	37.45%
5	SmartSet suggested	Prebuilt order set suggested	Event Shown (1/0)	0.0124	0.2182	0.0156	0.0092	Positive	6.03%	43.48%
6	Results reviewed in IB	Test result reviewed in inbox	Average Time Per Event (sec)	-0.0094	0.1403	-0.0074	-0.0115	Negative	3.88%	47.35%
7	Number of Active Diagnoses	Number of Active Diagnoses	Covariate	0.0117	0.1385	0.0139	0.0095	Positive	3.83%	51.18%
8	Follow-up modified	Follow‑up plan changed	Average Time Per Event (sec)	0.0075	0.1252	0.0095	0.0055	Positive	3.46%	54.64%
9	SmartSets activity selected	Order‑set workspace opened	Average Time Per Event (sec)	0.0143	0.1077	0.0162	0.0124	Positive	2.98%	57.62%
10	Patient is Hispanic/Latinx	Patient is Hispanic/Latinx	Covariate	0.0035	0.1002	0.0056	0.0013	Positive	2.77%	60.39%

In the pre-decision sensitivity model that truncated encounters at the point of the statin decision for initiated visits and at the first orders screen (or a matched median time) for non-initiated visits, cognitive load features before the decision point explained 62.98% of the model’s predictions, and discrimination improved (AUROC 0.95). Within this pre-decision window, average time per event still contributed over half of the total SHAP magnitude (53%) but shifted toward more negative mean SHAP values overall, whereas the proportion of contribution from event presence indicators (21%) and loop counts (17%) increased, with these features showing predominantly positive mean SHAP values. Encounters with more loops and longer time spent updating diagnosis-linking actions had the largest positive contributions (together ~ 20% of the top 10 contributions), indicating that repeated, slower work to link diagnoses to orders or visits was strongly associated with higher model-predicted probability of statin initiation. Longer time spent when a SmartSet was suggested, greater exposure to practice advisories, the SmartSet suggestion flag itself, more loops viewing the patient summary (“Storyboard”), and having the visit pharmacy modified also showed positive mean SHAP values, suggesting that richer, more deliberate use of structured tools and decision supports before the order screen is reached tends to be associated to encounters where statins are initiated. In contrast, longer time spent viewing past medication and order histories in a patient’s past history (“Chart Review”), time in the SmartSet activity workspace, and a higher number of active diagnoses had negative mean SHAP values, indicating that extensive retrospective review, time “parked” in the order set workspace without necessarily executing an order, and greater problem list burden were associated with lower model-predicted initiation. The one patient covariate of the top 10 significant contributors to the model’s prediction was the number of active diagnoses, where more diagnoses were more associated with lower model-predicted probability of statin initiation. The top 10 features are reported in Table 8, and all significant features are reported in Table A.6.

**Table 8:** Top 10 cognitive load features associated with Statin initiation when examining pre-statin initiation EHR audit log behavior versus truncated EHR audit log windows in non-statin initiation encounters

Rank	Metric Name	Plain Language Explanation	Cognitive Load Metric	Mean SHAP Value	Global Mean SHAP Value	95% CI Lower	95% CI Upper	Contribution Direction	Relative Contribution
1	Diagnosis association updated	Diagnosis linked/updated for an order or visit	Number of Loops	0.1286	0.4635	0.1414	0.1158	Positive	10.51%
2	Diagnosis association updated	Diagnosis linked/updated for an order or visit	Average Time Per Event (sec)	0.0134	0.4166	0.0212	0.0056	Positive	9.45%
3	SmartSet suggested	Prebuilt order set suggested	Average Time Per Event (sec)	0.0179	0.3441	0.0234	0.0123	Positive	7.80%
4	Chart Review Order report viewed	Past medication/orders history viewed	Average Time Per Event (sec)	-0.0842	0.3198	-0.0752	-0.0932	Negative	7.25%
5	SmartSets activity selected	Order set workspace opened	Average Time Per Event (sec)	-0.0179	0.3117	-0.0121	-0.0237	Negative	7.07%
6	Number of Active Diagnoses	Number of Active Diagnoses	Covariate	-0.0211	0.3113	-0.0157	-0.0265	Negative	7.06%
7	OurPractice Advisories displayed	Practice advisory alert shown	Average Time Per Event (sec)	0.0092	0.1927	0.0129	0.0054	Positive	4.37%
8	SmartSet suggested	Prebuilt order set suggested	Event Shown (1/0)	0.0046	0.1509	0.0070	0.0022	Positive	3.42%
9	Storyboard viewed	Sidebar patient summary viewed	Number of Loops	0.0034	0.1350	0.0058	0.0011	Positive	3.06%
10	Pharmacy for encounter modified	Visit pharmacy selected/changed	Event Shown (1/0)	0.0127	0.1315	0.0173	0.0080	Positive	2.98%

The total relative contribution of these features is 62.98% of the model’s predictions

Comparing the full‑encounter and pre‑decision models, we observed both consistent and shifting relationships between EHR activities, cognitive‑load metrics, and predicted statin initiation. In both models, event‑time features and a few covariates explained most of the summed absolute SHAP values, suggesting that workflow‑related cognitive load contributed more to model variation than measured case mix.

In the full‑encounter model, longer time editing the order list, using suggested SmartSets, modifying follow‑up plans, and opening the SmartSets workspace were positively associated with predicted initiation, whereas time spent on inbox tasks (messages, results) had negative associations; number of active diagnoses and Hispanic/Latinx ethnicity also showed positive effects. In contrast, the pre‑decision model highlighted loops and time spent updating diagnosis associations, engaging with suggested SmartSets and practice advisories, Storyboard loops, and pharmacy modifications as the strongest positive contributors, while reviewing past orders or medication histories, longer SmartSets workspace time, and higher active diagnoses had negative effects.

## Discussion

Overall, diagnosis linking, targeted use of prebuilt tools, and focused summary views characterized encounters with higher predicted initiation, whereas diffuse information‑seeking and higher problem‑list burden characterized those with lower predicted initiation. Though descriptive and non‑causal, these SHAP‑based patterns suggest that workflow elements like diagnosis association, prebuilt tool use, and historical data review may be useful targets for experimental redesigns.

### Summary

We identified statistically significant associations between cognitive load metrics and the likelihood of initiating statin therapy during primary care encounters. First, we observed that cognitive load does not have a linear relationship to statin initiation. Some types of cognitive load (in this study, engagement reflected by repetitive behaviors) suggest that a “middle ground” of provider effort is associated with a higher likelihood of statin initiation; that is, some engagement, but not too much or too little, is associated with more appropriate prescribing. We also found that under-engagement (few interactions) and over-engagement (excessive interactions) were associated with differing likelihoods of satin initiation, possibly reflecting unmeasured factors such as encounter complexity or workflow variation. These results illustrate the dynamic nature of cognitive load in clinical environments. By applying machine learning techniques to EHR metadata, we identified behavioral patterns that may inform hypothesis-generating insights for future CDSS design – highlighting opportunities to better align EHR features with provider cognitive processes. These findings suggest that considering cognitive load in interface design could help generate testable hypotheses for improving decision support in real-world care [40]. 

### Implications

This study offers hypothesis-generating insights into how cognitive load in EHR workflows is associated with clinical decision-making and outlines a potential framework for exploring ways to support decision quality. By measuring cognitive load indicators associated with prescribing decisions, we highlight key workflow moments that may be useful targets for future CDSS evaluation. These findings lay the groundwork for developing and prospectively testing interventions aimed at reducing unnecessary complexity and supporting clinicians in following guideline-recommended therapies.

Our methodological approach may have both near-term and long-term benefits for health systems. In the near term, these associations can inform exploration of workflow patterns that may hinder effective decision support. Over the longer term, this approach may help health systems monitor and study provider behavior at scale, contributing to data-driven quality improvement initiatives. These methods can also be refined to distinguish between cognitive load that are potentially helpful or unhelpful for providers, generating new hypotheses about how digital workflows shape clinical behaviors. For patients, improved prescribing practices from interventions testing such hypotheses can have a direct and meaningful impact on outcomes, particularly by increasing the use of appropriate statins to lower primary ASCVD risk.

The developed methodological framework is scalable and adaptable across clinical settings and EHR systems. Our data processing and workflow characterization techniques can support further research to enhance clinical decision support and promote evidence-based care. For example, the approach to linking audit log metadata clinical data can be adapted to address other behavior-related questions, such as specialty referrals, shared decision-making, and screening practices. Similarly, the identification of behaviors within the EHR and construction of cognitive load metrics are EHR-agnostic, relying on generalizable process mining and data science techniques. Together, these analytical methods provide a systematic way to evaluate associations between cognitive load, EHR use, and clinical behaviors, informing iterative design and evaluation of decision support tools.

### Limitations

Several limitations should be considered when interpreting our findings. First, the cognitive load and workflow measures used in this study were intentionally simple. While more sophisticated process mining techniques, such as clustering workflow sequences or calculating transition probabilities between EHR events, could yield deeper insights, we prioritized interpretable, actionable metrics aligned with behavior change theory. These simpler measures are more readily translatable into EHR tools intended to support clinical decision-making. Moreover, the application of EHR metadata for healthcare quality improvement remains an emerging area within clinical informatics. There is an ongoing need for standardization of EHR metadata metrics by organizations such as the Office of the National Coordinator for Health Information Technology (ONC), which could also enable validation of more complex measures through direct observation or qualitative research [41]. 

Second, our findings are based on data from a single EHR platform within one academic health center. While the specific events captured may vary across institutions, our computational approach was designed to be generalizable by focusing on abstracted, system-agnostic event types. We did not model the whole sequence of EHR actions (e.g., via recurrent or sequence-to-sequence models), as these sequence-based representations are often vendor- and site-specific, limiting generalizability across implementations [42]. Our focus on event-level cognitive load metrics supports comparability across encounters and sites. Future work could extend these methods to sequence-aware models within health systems to inform local quality improvement and user-experience design. While implementation may require adaptation to local data structures and clinical contexts, the overall methodology should be transferable to other EHR platforms.

Third, the cognitive load metrics used in this study do not distinguish between intrinsic, extrinsic, and germane cognitive load. As a result, we cannot determine whether the observed metrics primarily reflect the inherent complexity of the clinical decision (intrinsic load), inefficiencies introduced by the interface or workflow (extrinsic load), or the effort invested in integrating information for decision-making (germane load). Without this differentiation, design implications should be interpreted cautiously, as reducing extraneous demands could inadvertently diminish beneficial cognitive effort. Future work employing complementary methods such as task analysis, think-aloud protocols, or real-time workload assessment could help disentangle these components and inform more tailored design hypotheses for CDSS and EHR optimization [43]. 

Fourth, our SHAP-based interpretability analysis has important limitations. SHAP values quantify the contribution of model features to predictions within the trained XGBoost model, but they do not establish causal relationships between EHR events and statin initiation. Prior theoretical work has shown that SHAP and related explainability methods can misattribute importance when predictors are correlated or confounded and when causal structures are unspecified [44]. In this study, SHAP results are therefore interpreted as associative and hypothesis-generating, intended to identify potential workflow leverage points for future prospective and interventional testing rather than to imply that any specific event causes statin initiation.

Fifth, our analytical approach did not link individual EHR actions to specific medications, laboratory tests, or diagnoses. This design allowed for broad comparison across encounters with and without statin initiation. Future work could refine this approach by focusing on encounters involving shared medications or tests and by examining how cognitive effort on specific actions—such as reviewing cholesterol results or adjusting a statin order—relates to prescribing decisions.

Sixth, this study focused on a single clinical use case: initiating maintenance medications (statins) in primary care. Although our cohort identification and data linkage methods were tailored to this context, the overall approach may be applicable to other clinical domains. Importantly, the metadata metrics were derived independently of specifical clinical content, supporting broader adaptability. With further development, EHR vendors and health systems could aggregate and standardize such metrics to study workflow efficiency and decision support across varied clinical scenarios. For example, the study’s scope was intentionally limited to initiation and did not include decisions regarding statin titration. Further research can elaborate on how to differentiate the cognitive load of workflows for initiation versus titration, given the distinct behavioral mechanisms and decision-making processes associated with each. Finally, while our analyses focused on provider behavior, prior research suggests that patient hesitancy also shapes statin initiation [45]. The methods presented here could be extended to patient-facing interfaces and portals to better understand and enhance the digital patient experience.

## Conclusion

Our findings indicate that the likelihood of initiating statins is not simply determined by the amount of effort in the EHR but is associated with a nonlinear relationship between cognitive load and decision-making. Both lower and higher levels of engagement with the EHR were associated with reduced likelihood of initiating appropriate therapy, suggesting that an intermediate level of engagement may be conducive to guideline-concordant prescribing. Similarly, extreme engagement behaviors in the EHR can be associated with appropriate clinical actions, whereas an intermediate level of behavior may be associated with low engagement. By characterizing specific EHR interaction patterns and activity levels linked to statin initiation, this study identifies potential areas where decision support and workflow design could be prospectively tested to better align cognitive demands with effective clinical action.

More broadly, this study demonstrates the feasibility of deriving interpretable cognitive load metrics from EHR metadata and linking them to observed clinical behaviors. This methodological contribution provides a framework for generating hypotheses about how cognitive processes unfold in digital clinical environments. Continued research applying and refining these methods can support future efforts to design, evaluate, and iteratively improve decision-support interventions that help clinicians deliver care consistent with evidence-based guidelines.

## Supplementary Information

Below is the link to the electronic supplementary material.

Supplementary Material 1

## Data Availability

Due to the use of patient data in this study, deidentified versions of the data can be made available upon reasonable request. Dr. Safiya Richardson can be contacted at [Safiya.richardson@nyulangone.org](mailto: Safiya.richardson@nyulangone.org) for data requests.

## Abbreviations

Atherosclerotic cardiovascular disease (ASCVD): Condition caused by plaque buildup (atherosclerosis) in the arteries
ASCVD 10-year risk score: Estimate of a person’s chance of having an ASCVD event (e.g., heart attack or stroke) within the next 10 years
Clinical Decision Support Systems (CDSS): Tools or systems within the EHR that provide clinicians with information, alerts, or recommendations to improve patient care decisions
Cognitive Load: The mental effort required to process information and complete tasks, influenced by the complexity of the information, the environment, and the interface design
Electronic Health Record (EHR): A digital system for storing and managing patient medical records and clinical workflows
EHR Metadata: Data automatically generated and stored by the EHR that describes user interactions, such as keystrokes, clicks, screen time, and navigation patterns
Gradient-boosted forests: Machine learning method that combines decision trees built in sequence in which new trees correct errors of previous trees to improve accuracy
Hadoop distributed file system: System for storing large data sets across multiple machines, allowing processing in parallel
Hawthorne Effect: When people change their behavior because they are aware they are being observed
AUROC (Area Under the Curve – Receiver Operating Characteristic): A measure of a binary classifier’s performance; higher values indicate better discrimination between positive and negative cases
Random Forest: A machine learning method that uses multiple decision trees to make predictions or classifications; in this study, used to predict statin initiation from cognitive load metrics
Shapley additive explanation (SHAP) values: Method to explain machine learning model’s predictions by quantifying each feature’s contribution to a specific prediction
Statin: A class of cholesterol-lowering medications used to reduce cardiovascular risk, often prescribed for patients with elevated ASCVD risk, by blocking the production of low-density lipids (LDL) in the liver, helping prevent heart attacks and strokes
STROBE: The Strengthening the Reporting of Observational Studies in Epidemiology (STROBE) guidelines for reporting observational studies
Tukey IQR rule: A statistical method for identifying outliers by defining data points lying more than 1.5 times the interquartile range above the third quartile or below the first quartile
